# Supplementary material for: Comprehensive proteomics and meta-analysis of COVID-19 host response
Source: Nat Commun. 2023 Sep 22;14:5921. doi: 10.1038/s41467-023-41159-z (PMC10516886; doi:10.1038/s41467-023-41159-z)
Supplement: Supplementary file 1 — Supplementary Information [file 41467_2023_41159_MOESM1_ESM.pdf]

Supplementary Figures

for manuscript:

Comprehensive proteomics and meta-analysis of COVID-19 host response

Authors:

Haris Babačić[1§], Wanda Christ[2], José Eduardo Araújo[1], Georgios Mermelekas[1], Nidhi Sharma[1], Janne Tynell[2], Marina García[2], Renata Varnaite[2], Hilmir Asgeirsson[3,4], Hedvig Glans[2,3], Janne Lehtiö[1], Sara Gredmark Russ[2,3,5] , Jonas Klingström[2,6*], Maria Pernemalm[1*§]

Affiliations:

[1] Science for Life Laboratory and Department of Oncology and Pathology, Karolinska Institute, Stockholm, 171 77, Sweden

[2] Centre for Infectious Medicine, Department of Medicine Huddinge, Karolinska Institute, Stockholm, 171 77, Sweden

[3] Department of Infectious Diseases, Karolinska University Hospital, 141 86 Stockholm, Sweden

[4] Unit of Infectious Diseases, Department of Medicine Huddinge, Karolinska Institute, Stockholm, 141 86, Sweden

[5] The Laboratory for Molecular Infection Medicine Sweden (MIMS), Umeå, Sweden

[6] Division of Molecular Medicine and Virology (MMV), Department of Biomedical and Clinical Sciences (BKV), Linköping University, 581 83 Linköping, Sweden

* These authors jointly supervised this work

[§] Corresponding authors: haris.babacic@ki.se; maria.pernemalm@ki.se;

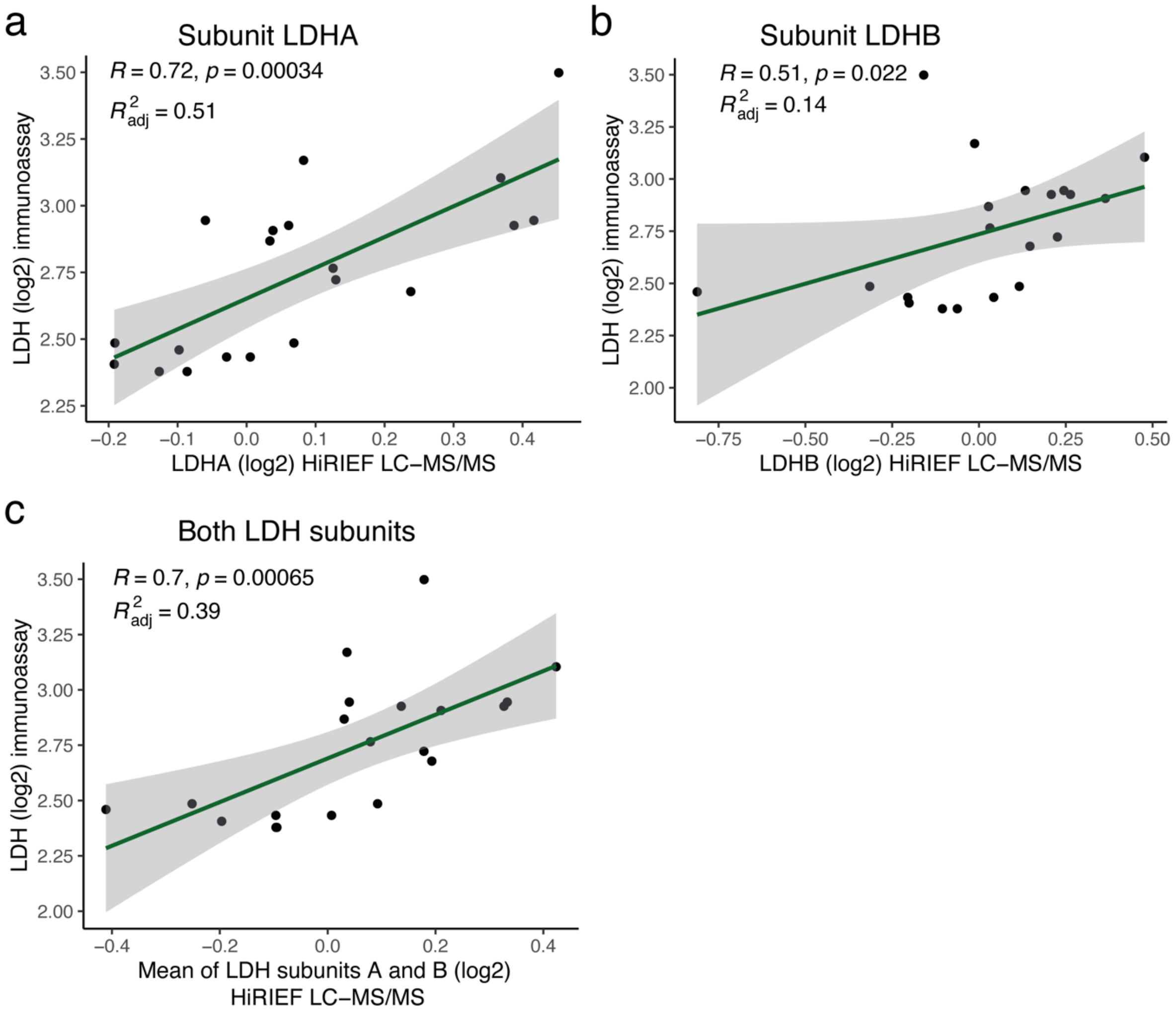

Figure S1. Agreement in quantifying lactate dehydrogenase (LDH) between HiRIEF LC-MS/MS and clinical chemistry assays (log2-values): a. Subunit A of LDH identified by HiRIEF LC-MS/MS compared to LDH immunoassay; **b.** Subunit B of LDH identified by HiRIEF LC-MS/MS compared to LDH immunoassay; **c.** LDH levels quantified as the average of levels of subunit A and B compared to LDH immunoassay. Worth noting is that the clinical assays quantify the canonical LDH protein as a whole protein, more specifically a particular epitope, whereas HiRIEF LC-MS/MS differentiated between the LDH protein subunits A and B. The line represents the linear regression fit line and the surrounding shaded area 95% CI. The p values for the Spearman correlation coefficients (*R*) were obtained with a two-sided t test.

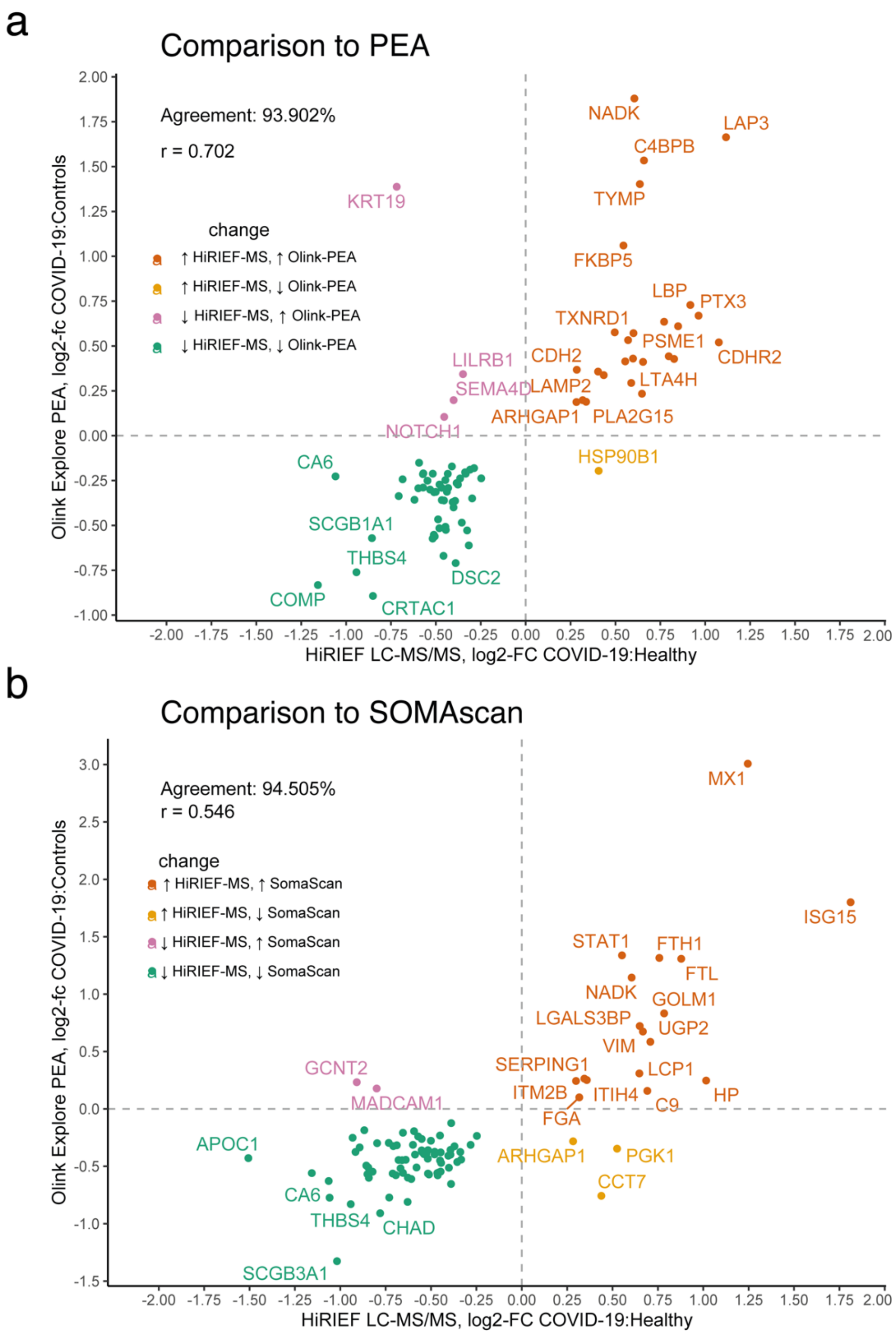

Figure S2. Differentially altered plasma proteins (n = 463) in COVID-19 as compared to healthy controls identified by HiRIEF LC-MS/MS, adjusted for age, sex, hypertension, and diabetes with limma models (and two-sided modified t statistic), in comparison to: **a.** Differentially altered plasma proteins in COVID-19 identified by PEA, adjusted for age, sex, ethnicity, heart disease, diabetes, hypertension, hyperlipidaemia, pulmonary disease, kidney disease, and immuno-compromised status as covariates; **b.** Differentially altered plasma proteins in COVID-19 identified by SOMAscan, adjusted for age and sex.

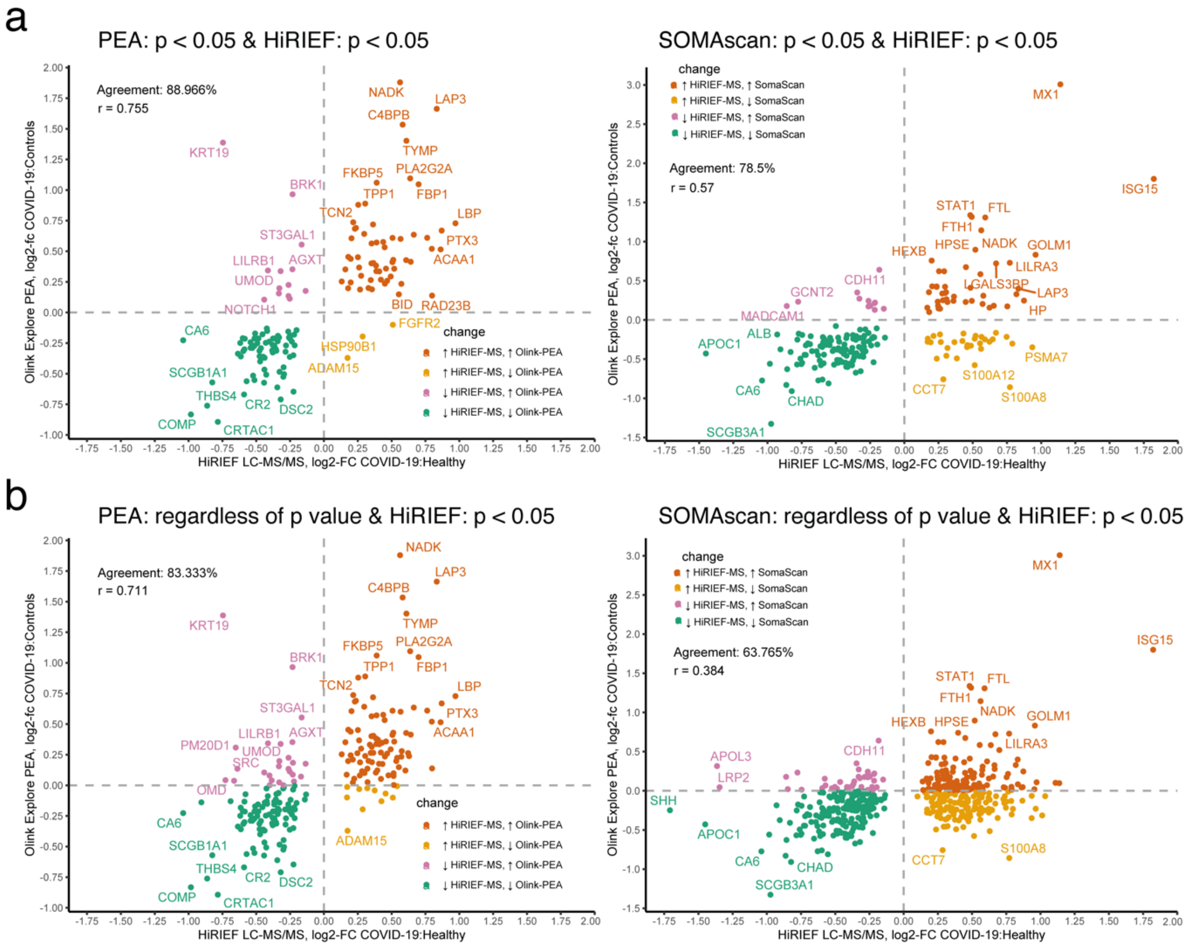

Figure S3. Sensitivity analysis on the agreement between HiRIEF LC-MS/MS analyses of COVID-19 plasma proteome alterations (at $p < 0.05$) and analyses by PEA and SOMAscan: a. Comparison of protein alterations identified by HiRIEF LC-MS/MS to proteins altered in COVID-19 at $p < 0.05$, no FDR, by PEA (left) and SOMAscan (right); **b.** Comparison of protein alterations identified by HiRIEF LC-MS/MS to statistically significant and non-significant proteins altered in COVID-19, by PEA (left) and SOMAscan (right). The agreement is represented as proportion (in %) of proteins changing in the same direction out of the total number of overlapping proteins and with Spearman's correlation coefficient (r)

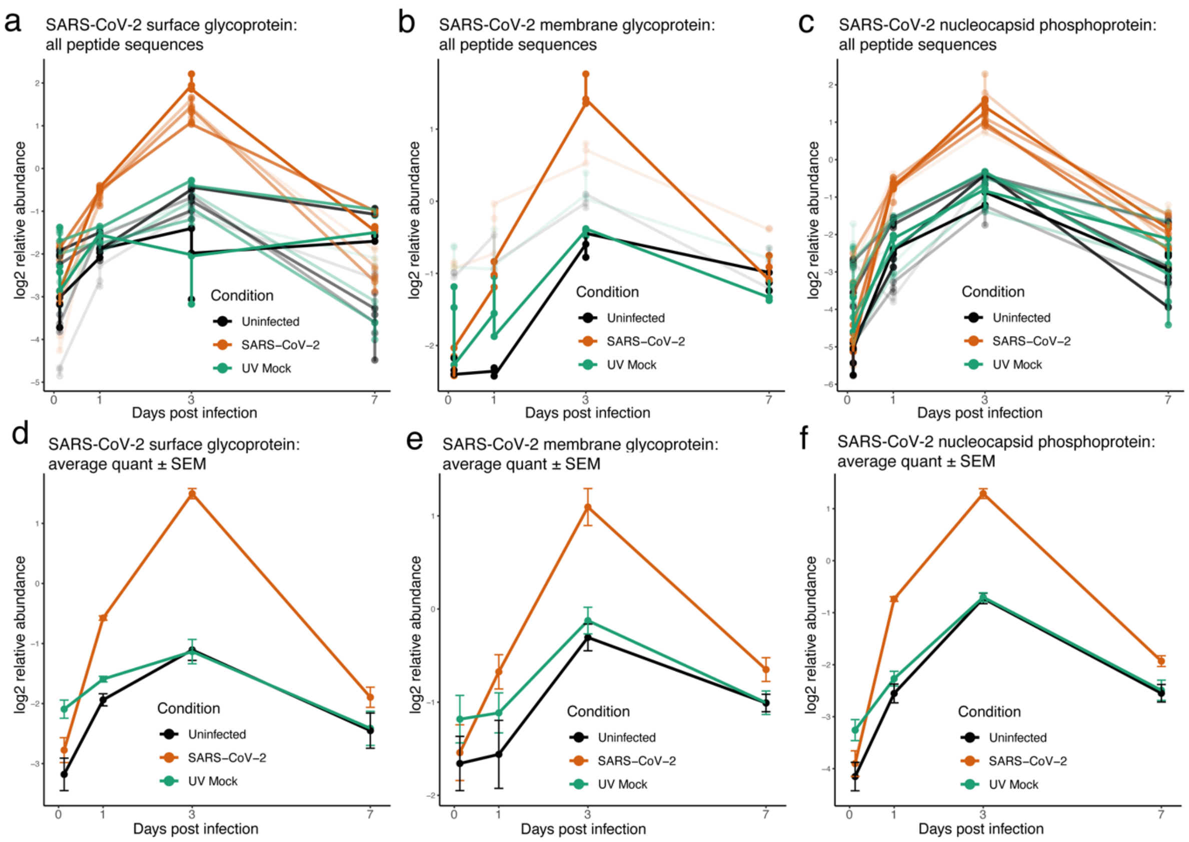

Figure S4. SARS-CoV-2 viral protein levels in infected cell lines at different time points, as compared to non-infected and UV-mock treated cells. a-c. Variance in the levels of all protein sequences (codon-centric) mapping to a specific viral protein over time; **d-f.** Estimated protein levels presented as means (dots) and standard error of the mean (SEM, error bars) of the quantifications. All cells in all conditions were run in biological replicates (n = 3 each).

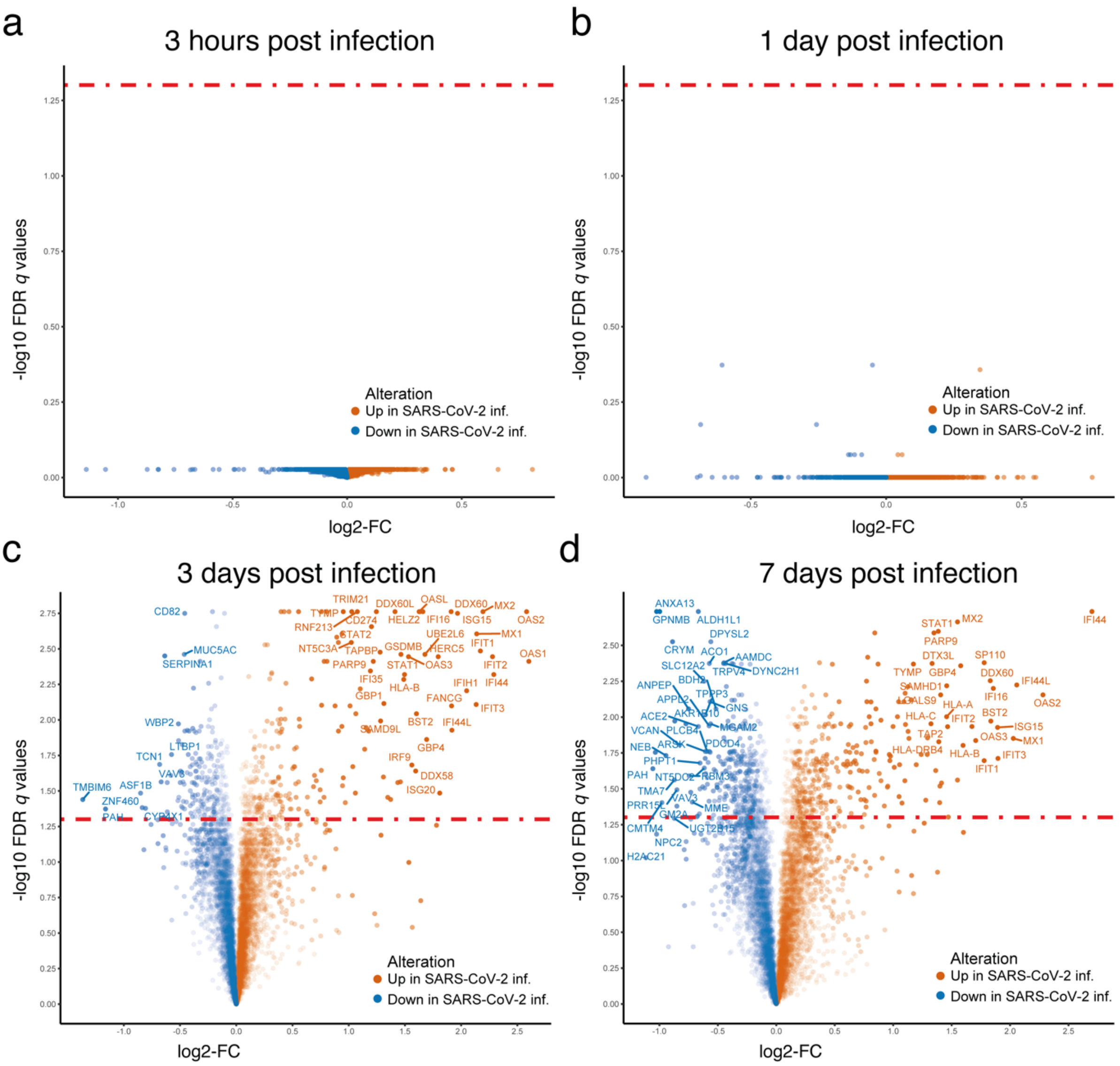

Figure S5. Volcano plots for the comparison SARS-CoV-2-nfected vs non-infected Calu-3 cells at different time points after infection: a. 3 hours; b. 1 day; c. 3 days; d. 7 days. The log2-fold change (log2-FC) refers to a log2 mean difference between infected and non-infected cells at the respective time point. The -log10 FDR-adjusted p values (*q* values) are plotted on the y axis and based on a two-sided t test. The horizontal dashed lines show the threshold of statistical significance ($\alpha = 0.05$).

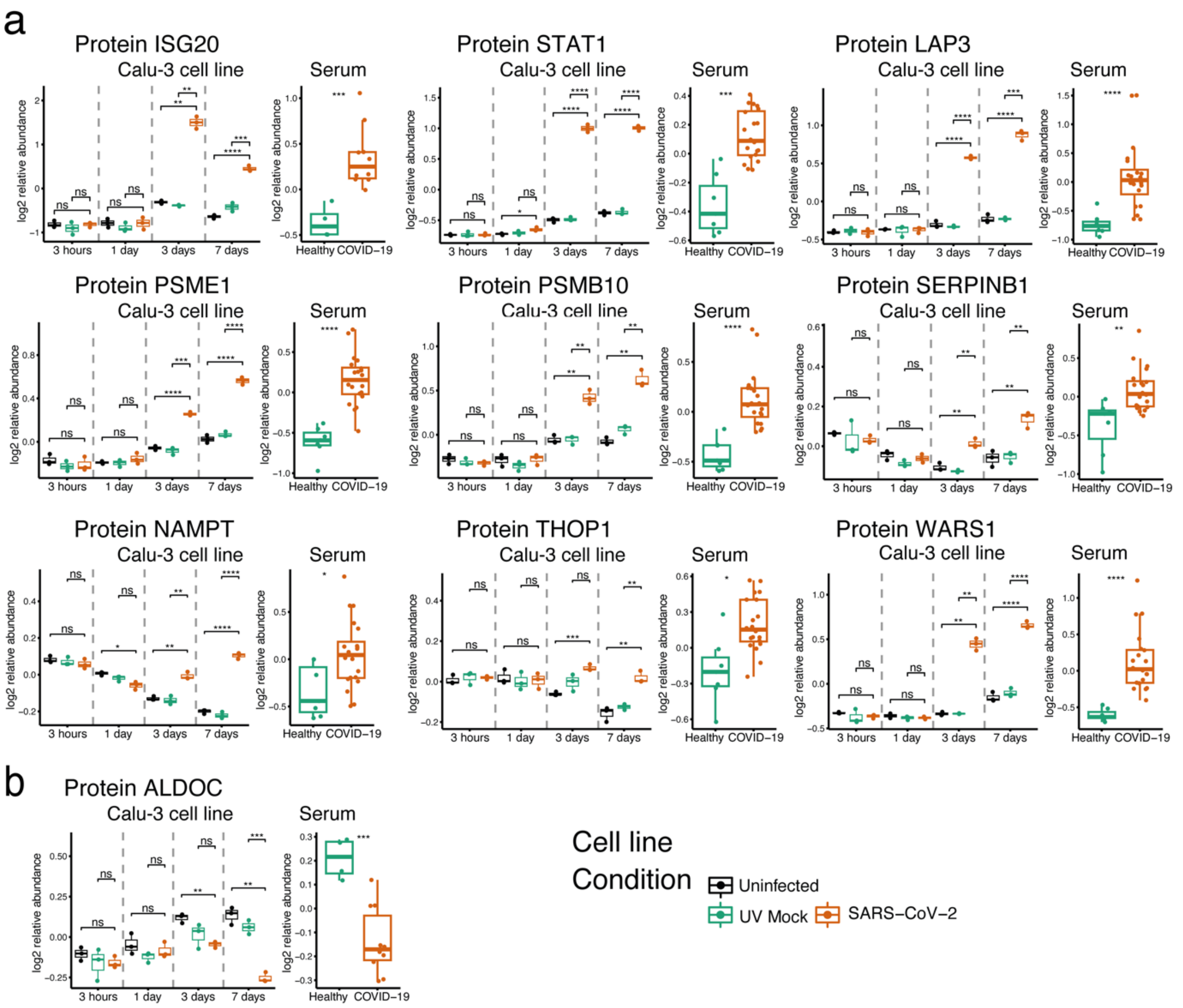

Figure S6. Boxplots for the remaining proteins consistently upregulated (a) and downregulated (b) at both day 3 and day 7 post infection, and in serum of COVID-19 patients. The boxplots show protein levels in SARS-CoV-2-infected Calu-3 cells at different time points, and comparison of infected cells to non-infected cells and cells treated with UV-inactivated SARS-CoV-2. All cells in each condition were cultured as biological replicates (n = 3 each). The box centre represents the median, the lower and upper box limits the 25th and 75th percentile, respectively, and whiskers' limits the minimum and maximum values of the data after removing outliers. In addition, boxplot of serum levels of the respective protein in COVID-19 patients compared to healthy controls. Abbreviations: n.s. = not significant, * = p < 0.05, ** = p < 0.01, *** = p < 0.005, **** = p < 0.001. The p values were determined with a two-sided t test and adjusted for multiple testing with the FDR.

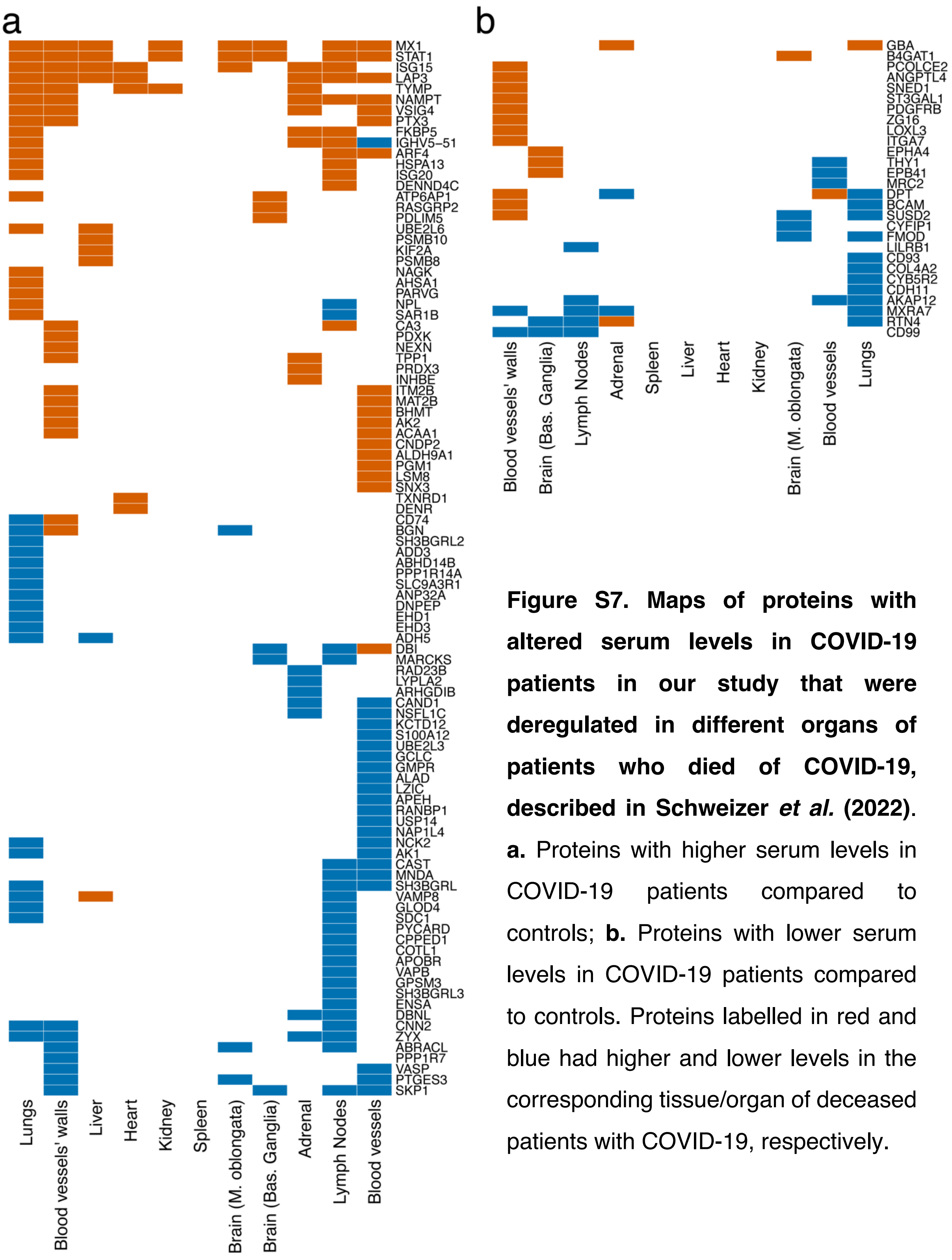

Figure S7. Maps of proteins with altered serum levels in COVID-19 patients in our study that were deregulated in different organs of patients who died of COVID-19, described in Schweizer *et al.* (2022). **a.** Proteins with higher serum levels in COVID-19 patients compared to controls; **b.** Proteins with lower serum levels in COVID-19 patients compared to controls. Proteins labelled in red and blue had higher and lower levels in the corresponding tissue/organ of deceased patients with COVID-19, respectively.

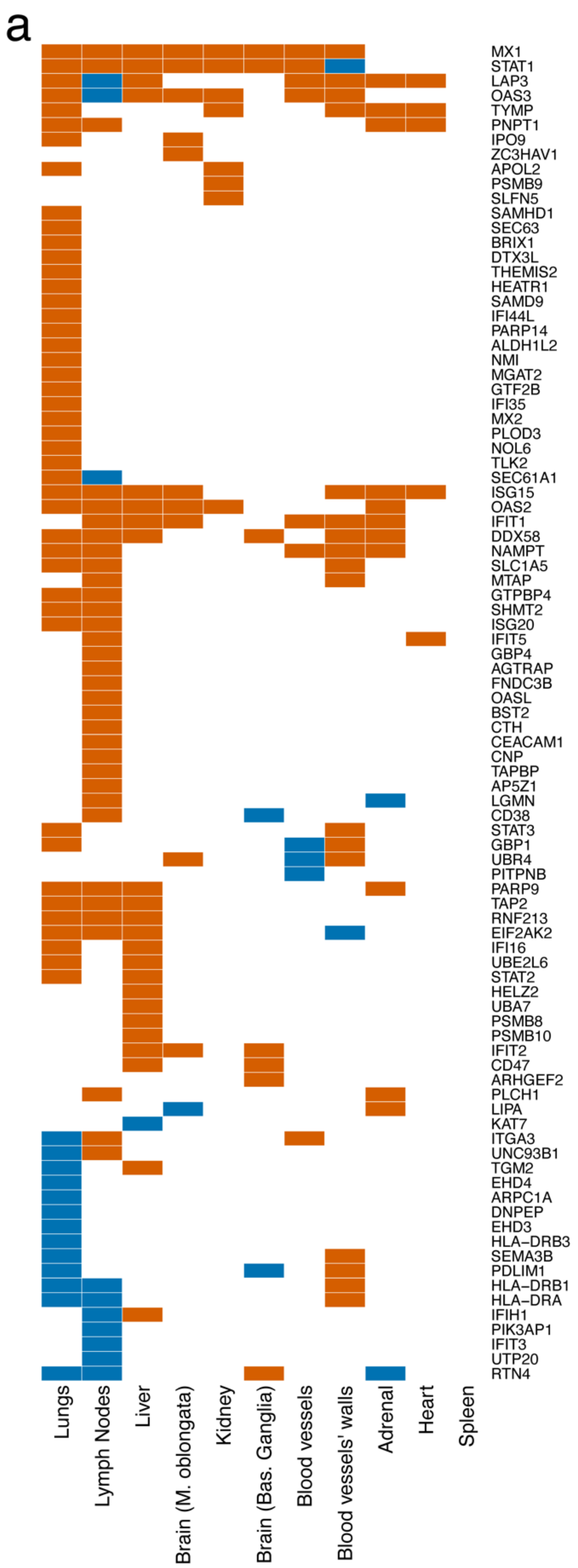

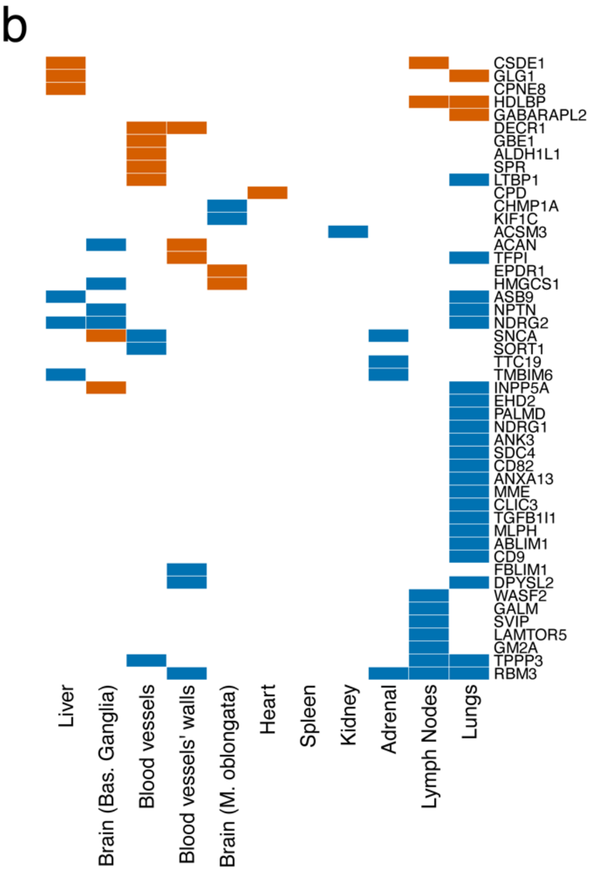

Figure S8. Maps of proteins with altered levels at Day 3 after infection in the Calu-3 cell line in our study that were deregulated in different organs of patients who died of COVID-19, described in Schweizer *et al.* (2022). **a.** Proteins with higher levels at Day 3 after infection as compared to non-infected controls; **b.** Proteins with higher levels at Day 3 after infection as compared to non-infected controls. Proteins labelled in red and blue had higher and lower levels in the corresponding tissue/organ of deceased patients with COVID-19, respectively.

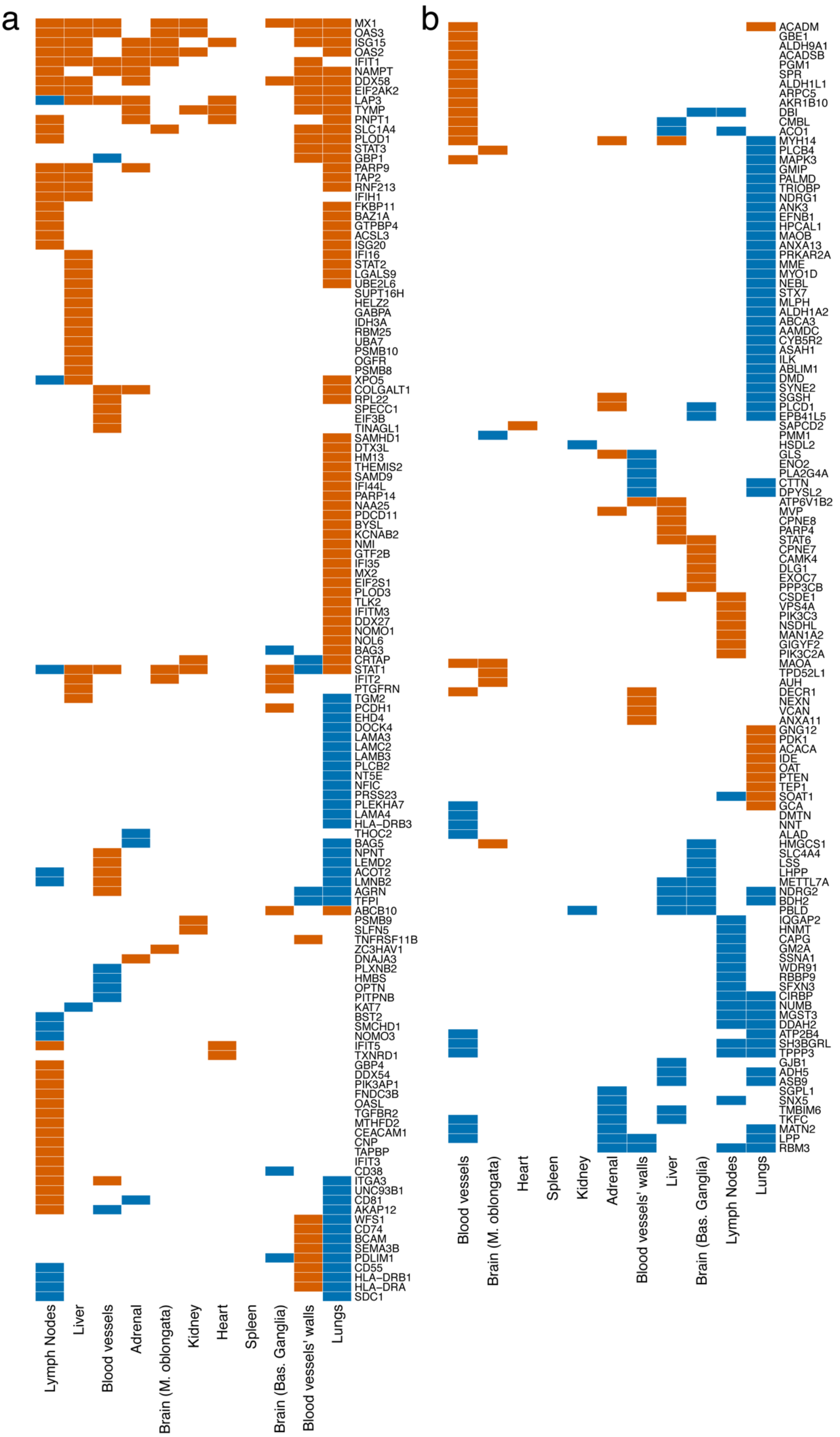
a
MX1
OAS3
ISG15
OAS2
IFIT1
NAMPT
DDX58
EIF2AK2
LAP3
TYMP
PNPT1
SLC1A4
PLOD1
STAT3
GBP1
PARP9
TAP2
RNF213
IFIH1
FKBP11
BAZ1A
GTPBP4
ACSL3
ISG20
IFI16
STAT2
LGALS9
UBE2L6
SUPT16H
HELZ2
GABPA
IDH3A
RBM25
UBA7
PSMB10
OGFR
PSMB8
XPO5
COLGALT1
RPL22
SPECC1
EIF3B
TINAGL1
SAMHD1
DTX3L
HM13
THEMIS2
SAMD9
IFI44L
PARP14
NAA25
PDCD11
BYSL
KCNAB2
NMI
GTF2B
IFI35
MX2
EIF2S1
PLOD3
TLK2
IFITM3
DDX27
NOMO1
NOL6
BAG3
CRTAP
STAT1
IFIT2
PTGFRN
TGM2
PCDH1
EHD4
DOCK4
LAMA3
LAMC2
LAMB3
PLCB2
NT5E
NFIC
PRSS23
PLEKHA7
LAMA4
HLA−DRB3
THOC2
BAG5
NPNT
LEMD2
ACOT2
LMNB2
AGRN
TFPI
ABCB10
PSMB9
SLFN5
TNFRSF11B
ZC3HAV1
DNAJA3
PLXNB2
HMBS
OPTN
PITPNB
KAT7
BST2
SMCHD1
NOMO3
IFIT5
TXNRD1
GBP4
DDX54
PIK3AP1
FNDC3B
OASL
TGFBR2
MTHFD2
CEACAM1
CNP
TAPBP
IFIT3
CD38
ITGA3
UNC93B1
CD81
AKAP12
WFS1
CD74
BCAM
SEMA3B
PDLIM1
CD55
HLA−DRB1
HLA−DRA
SDC1
Lymph Nodes
Liver
Blood vessels
Adrenal
Brain (M. oblongata)
Kidney
Heart
Spleen
Brain (Bas. Ganglia)
Blood vessels' walls
Lungs
b
ACADM
GBE1
ALDH9A1
ACADSB
PGM1
SPR
ALDH1L1
ARPC5
AKR1B10
DBI
CMBL
ACO1
MYH14
PLCB4
MAPK3
GMIP
PALMD
TRIOBP
NDRG1
ANK3
EFNB1
HPCAL1
MAOB
ANXA13
PRKAR2A
MME
MYO1D
NEBL
STX7
MLPH
ALDH1A2
ABCA3
AAMDC
CYB5R2
ASAH1
ILK
ABLIM1
DMD
SYNE2
SGSH
PLCD1
EPB41L5
SAPCD2
PMM1
HSDL2
GLS
ENO2
PLA2G4A
CTTN
DPYSL2
ATP6V1B2
MVP
CPNE8
PARP4
STAT6
CPNE7
CAMK4
DLG1
EXOC7
PPP3CB
CSDE1
VPS4A
PIK3C3
NSDHL
MAN1A2
GIGYF2
PIK3C2A
MAOA
TPD52L1
AUH
DECR1
NEXN
VCAN
ANXA11
GNG12
PDK1
ACACA
IDE
OAT
PTEN
TEP1
SOAT1
GCA
DMTN
NNT
ALAD
HMGCS1
SLC4A4
LSS
LHPP
METTL7A
NDRG2
BDH2
PBLD
IQGAP2
HNMT
CAPG
GM2A
SSNA1
WDR91
RBBP9
SFXN3
CIRBP
NUMB
MGST3
DDAH2
ATP2B4
SH3BGRL
TPPP3
GJB1
ADH5
ASB9
SGPL1
SNX5
TMBIM6
TKFC
MATN2
LPP
RBM3
Blood vessels
Brain (M. oblongata)
Heart
Spleen
Kidney
Adrenal
Blood vessels' walls
Liver
Brain (Bas. Ganglia)
Lymph Nodes
Lungs

Figure S9. Maps of proteins with altered levels at Day 7 after infection in the Calu-3 cell line in our study that were deregulated in different organs of patients who died of COVID-19, described in Schweizer *et al.* (2022). a. Proteins with higher levels at Day 7 after infection as compared to non-infected controls; **b.** Proteins with higher levels at Day 7 after infection as compared to non-infected controls. Proteins labelled in red and blue had higher and lower levels in the corresponding tissue/organ of deceased patients with COVID-19, respectively.

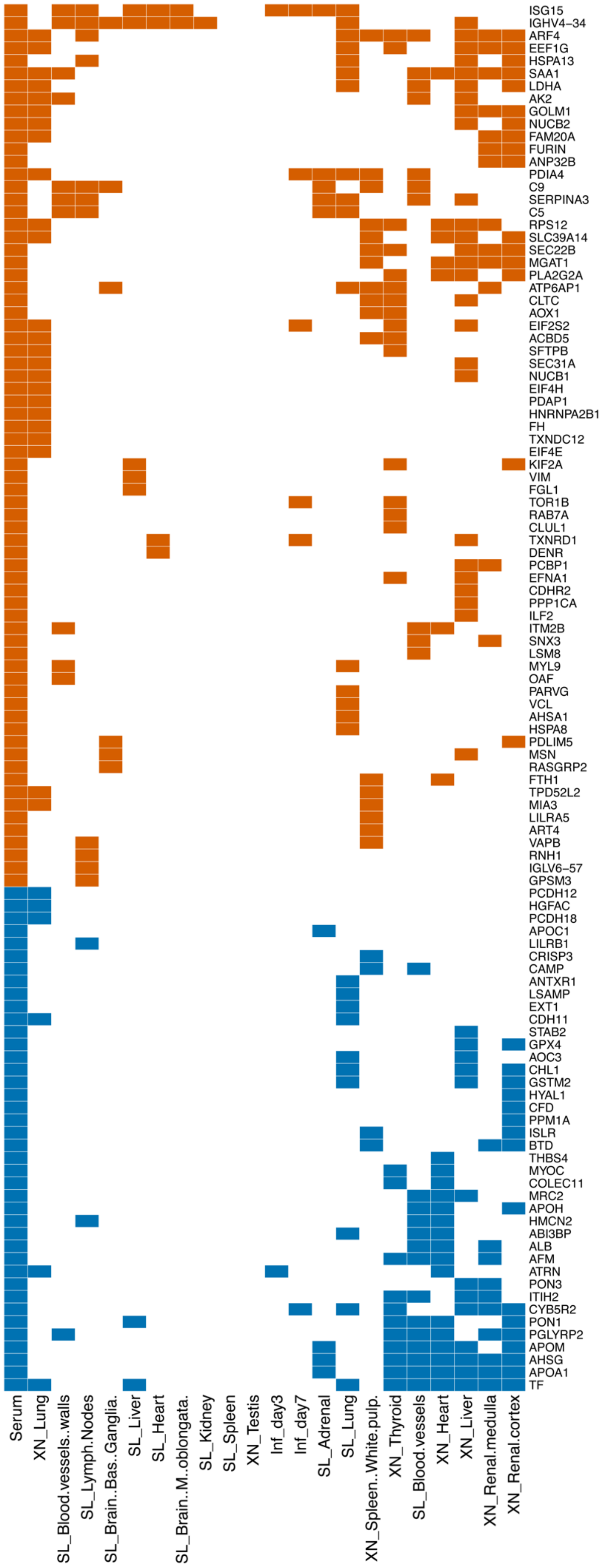

Figure S10. Maps of proteins with altered levels in the serum of COVID-19 patients in our study that were deregulated in the same direction in at least one organs of patients who died of COVID-19, described in Schweizer *et al.* (2022) datesets (annotated with SL) or the Xiu Nie (2021) datasets (annotated with XN). Proteins labelled in red and blue had higher and lower levels in the corresponding tissue/organ of deceased patients with COVID-19, respectively.

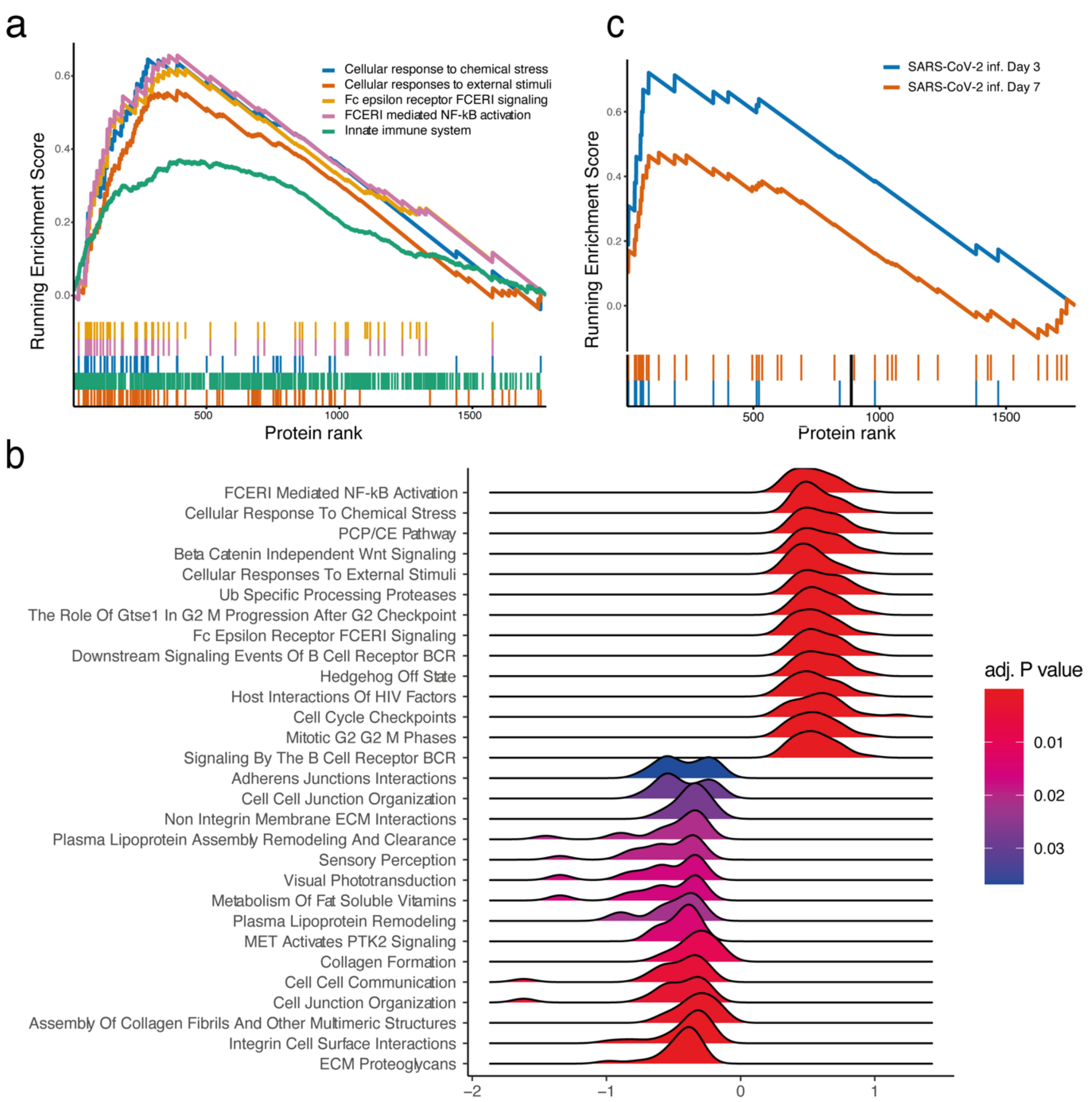

Figure S11. GSEA of COVID-19 serum proteome alterations: a. Top five enriched gene sets according to normalised enrichment score (NES) among REACTOME pathways at 5% FDR, permutation test. The rank (x axis) of the protein belonging in the gene set is based on log2-FC comparing mean serum levels between COVID-19 and PCR-negative healthy controls. The enrichment score is plotted on the y axis; **b.** Enriched REACTOME gene sets (x axis) with NES values (y axis) above 90% and below 10% quantile of NES; **c.** Enrichment of the protein sets upregulated in the SARS-CoV-2 infected cell lines at days 3 and 7, at 5% FDR and log2-FC > 0.5. The downregulated protein sets were not enriched in the serum.

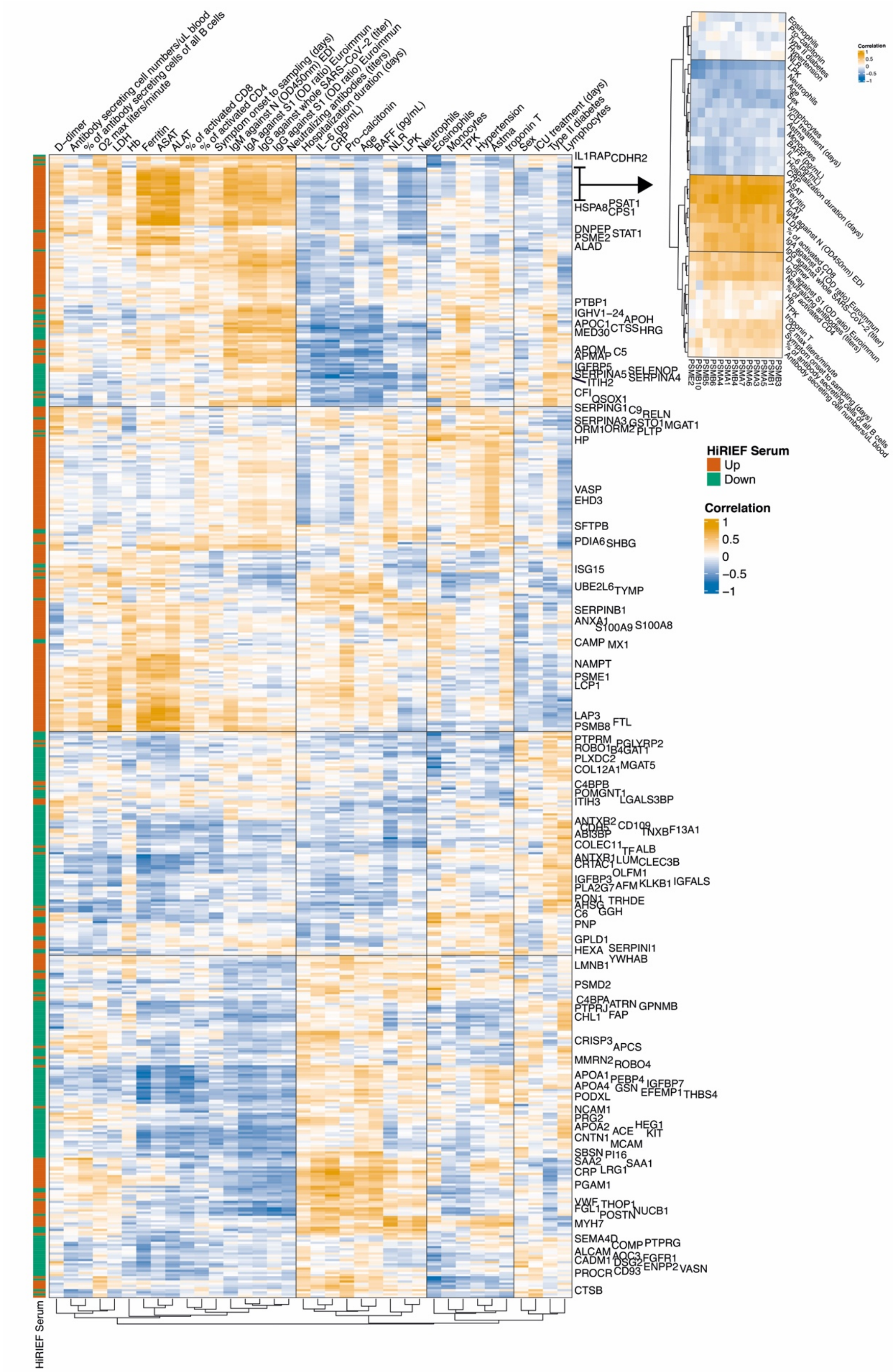

Figure S12. Correlation between plasma proteins and clinical parameters. The heatmap shows protein levels clustered with hierarchical clustering (distance: 1 – Spearman's ρ). It shows four clusters of clinical variables (x axis) and four clusters of proteins (y axis), separated

by the full black lines. A cluster of proteins (first from above) had a positive correlation between higher serum levels and immune response and a negative correlation with markers of severity, such as days of hospitalisation, CRP, IL6, and Pro-calcitonin. This cluster consisted of the majority of proteasomal proteins that are shown in the upper right corner. The cluster at the bottom had a positive correlation with markers of severity and negative correlation with immune response and included VIL1.

The row annotation “HiRIEF Serum” refers to the direction of the protein alteration in the serum of COVID-19 in our study.

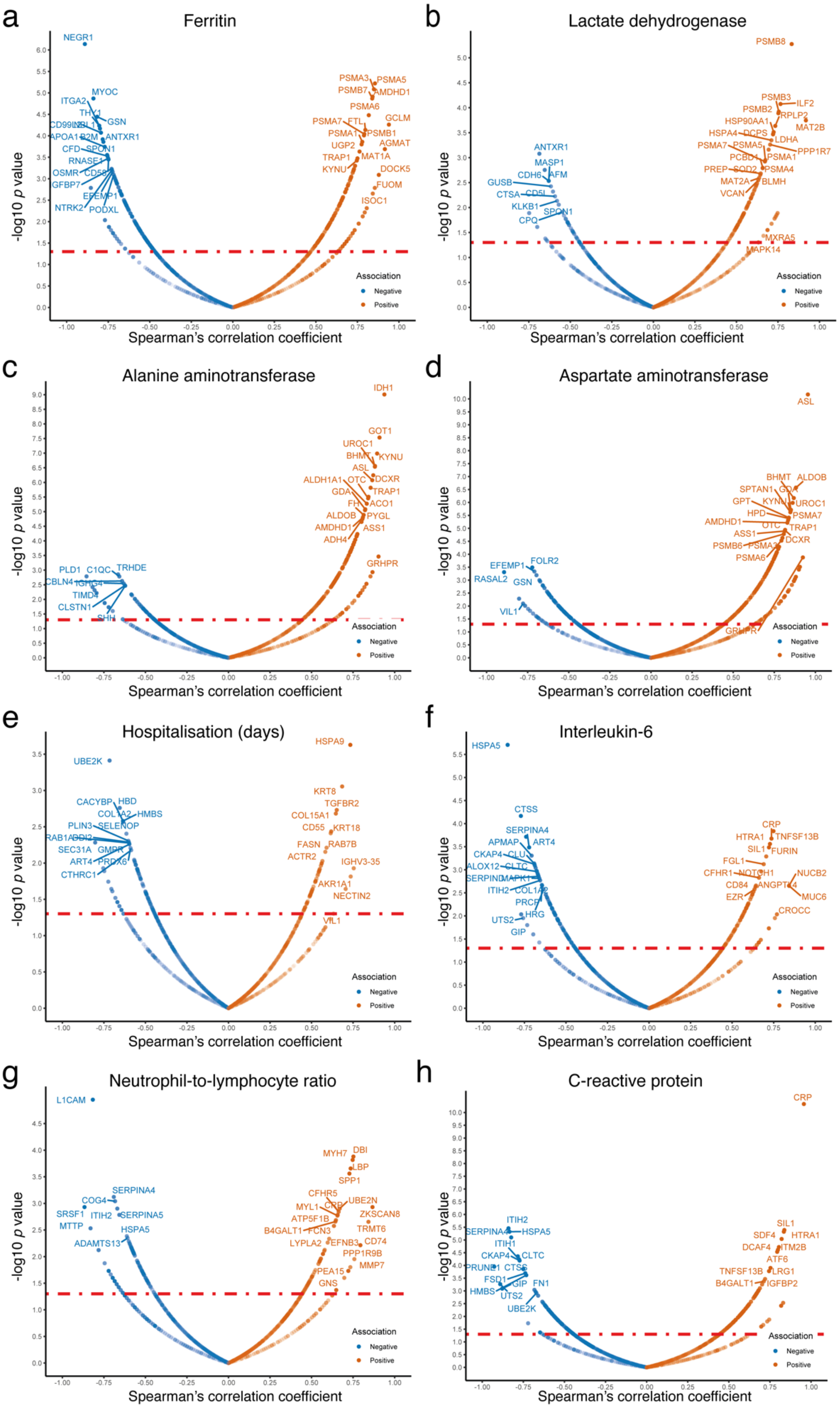

Figure S13. Volcano plots on correlation between clinical parameters and plasma proteins: a. Ferritin; **b.** LDH; **c.** AST; **d.** ALT; **e.** Hospitalisation; **f.** IL6; **g.** NLR; **h.** CRP.

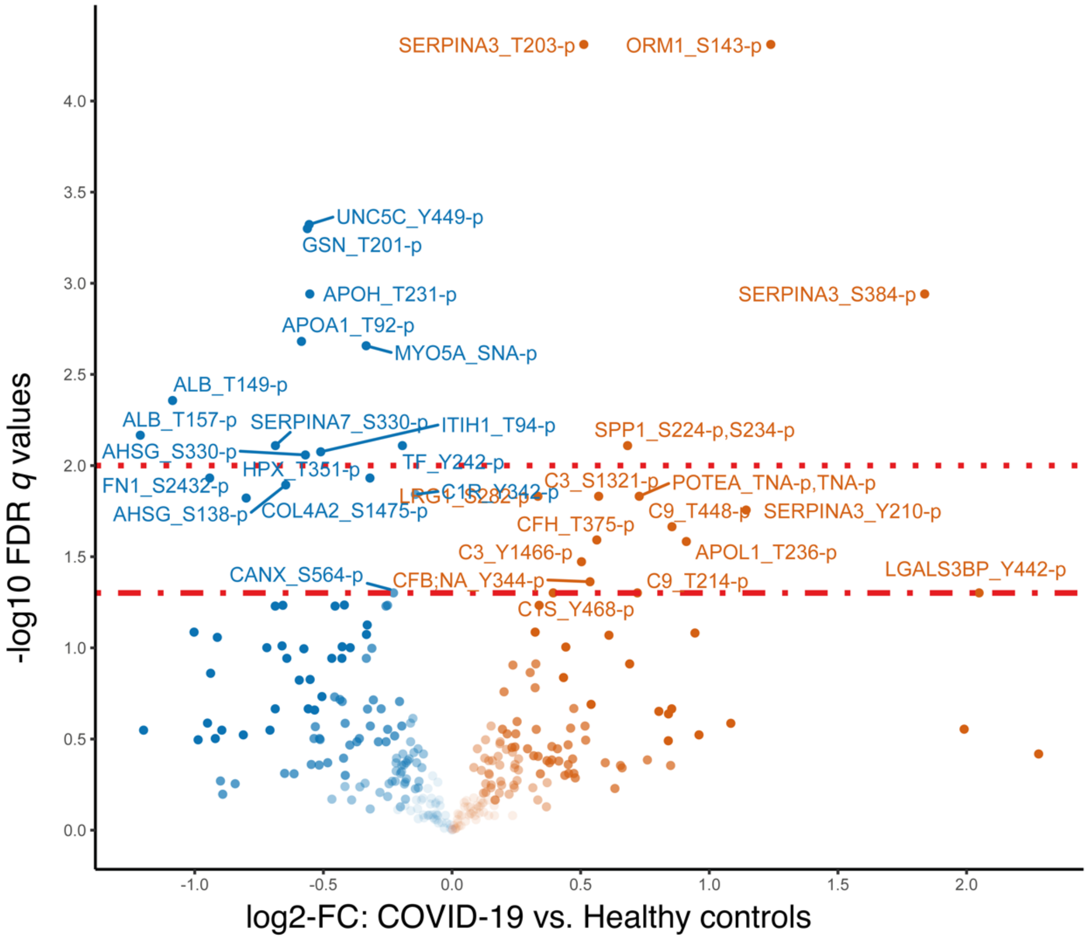

Figure S14. Volcano plot showing differentially altered proteins in serum of COVID-19 patients as compared to controls. The phosphosites are annotated according to the peptides' match to PhosphoSitePlus, mapped to the Uniprot canonical proteins. The dot-dashed lines indicated threshold of 5% FDR, whereas the dotted line indicates a threshold of 1% FDR.

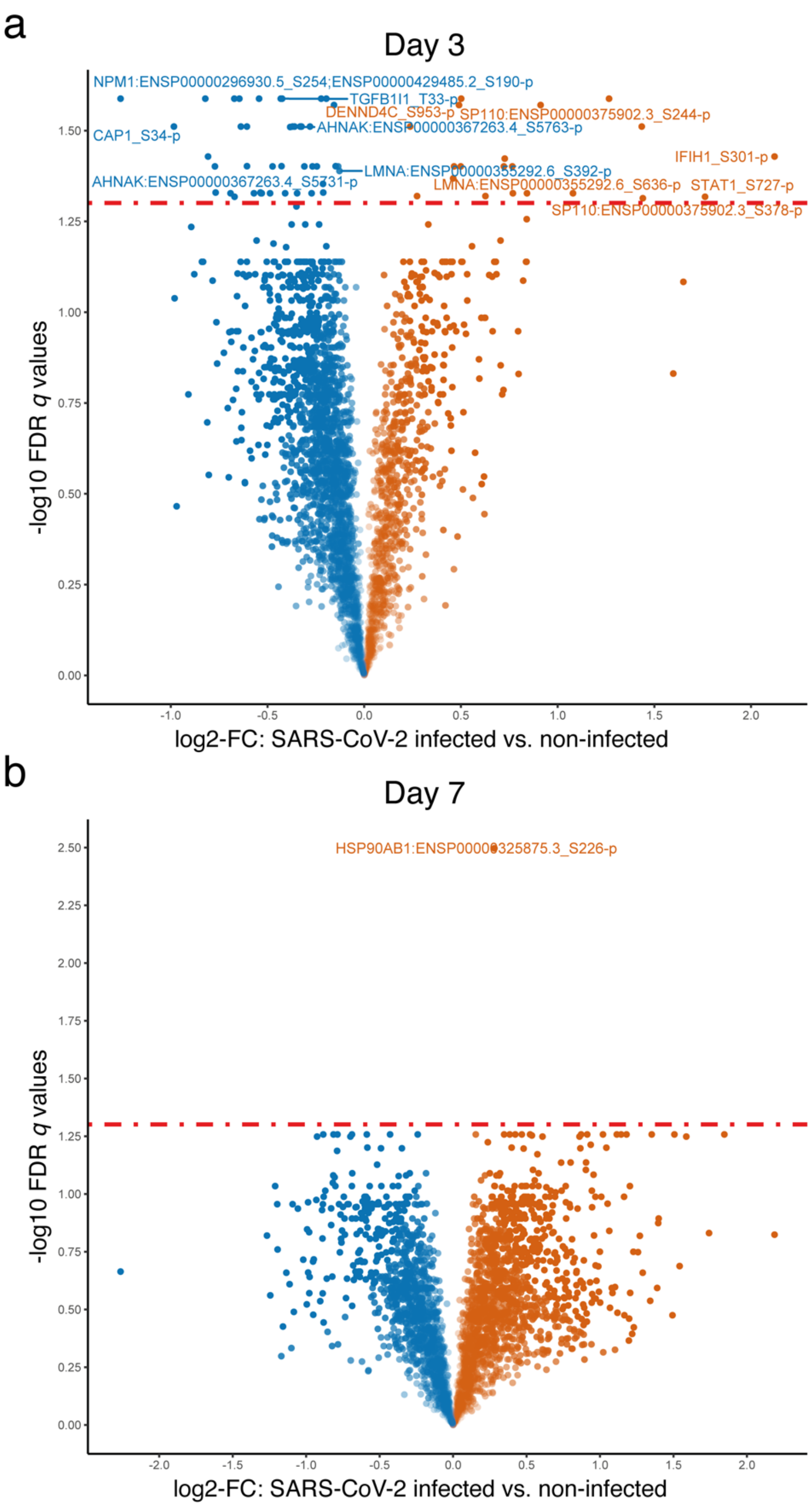

Figure S15. Differentially altered phosphosites in SARS-CoV-2-infected Calu-3 cells at: **a**. Day 3; and **b**. Day 7. The phosphosites are annotated according to the peptides' match to PhosphoSitePlus if found in the database, mapped to the Uniprot canonical proteins. Novel phosphosites are annotated with our pipeline, based on the ENSEMBL canonical proteins. The dot-dashed lines indicated threshold of 5% FDR, whereas the dotted line indicates a threshold of 1% FDR.

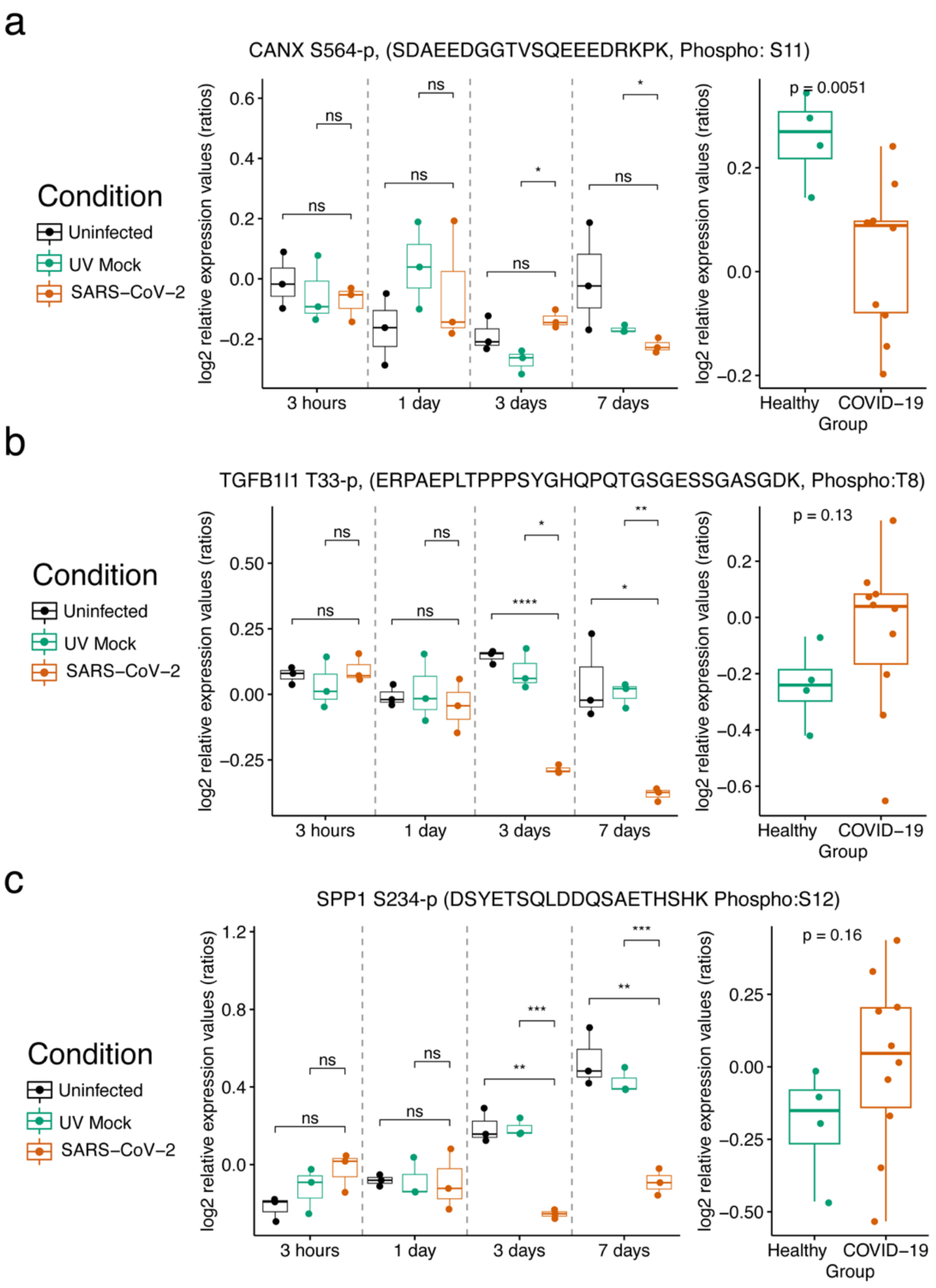

Figure S16. Boxplots showing levels of phosphorylated peptides in the SARS-CoV-2-infected cell lines and in serum of COVID-19 patients. a. CANX S564-p was the only phosphosite detected in the cell lines and serum that had a change in serum levels; **b.** and **c.** TGFB1I1 T33-p and SPP1 234-p did not have a change in serum levels but had a strong decrease in the cell lines 3- and 7- days after infection. All cells in each condition were cultured as biological replicates (n = 3 each). The box centre represents the median, the lower and upper box limits the 25th and 75th percentile, respectively, and whiskers' limits the minimum and maximum values of the data after removing outliers. Abbreviations: n.s. = not significant, * = p < 0.05, ** = p < 0.01, *** = p < 0.005, **** = p < 0.001. The p values were determined with a two-sided t test and adjusted for multiple testing with the FDR.

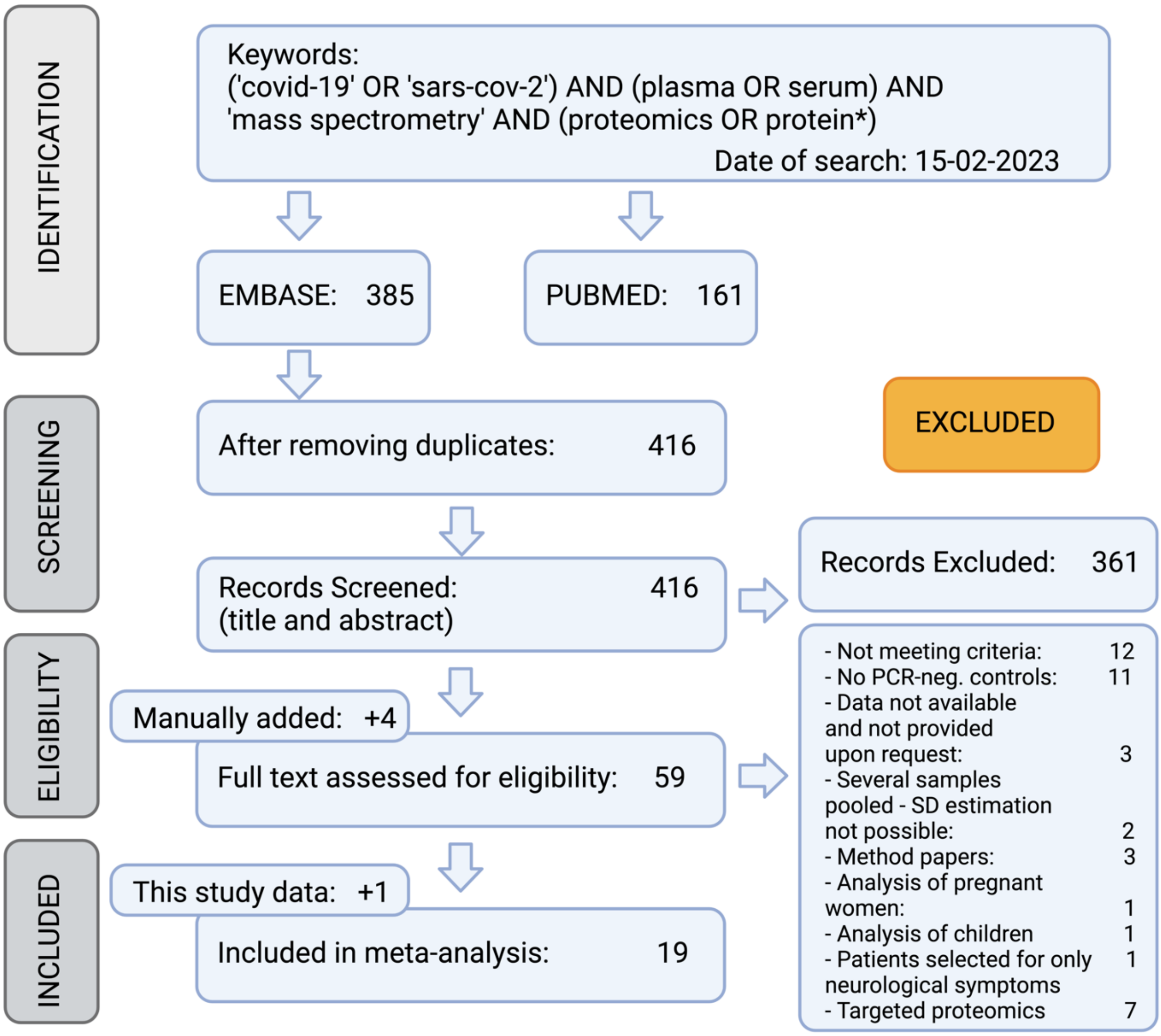

Figure S17. PRISMA systematic review workflow. Steps of the systematic review and the number of references obtained from database search, processed in the screening steps, and assessed for eligibility.

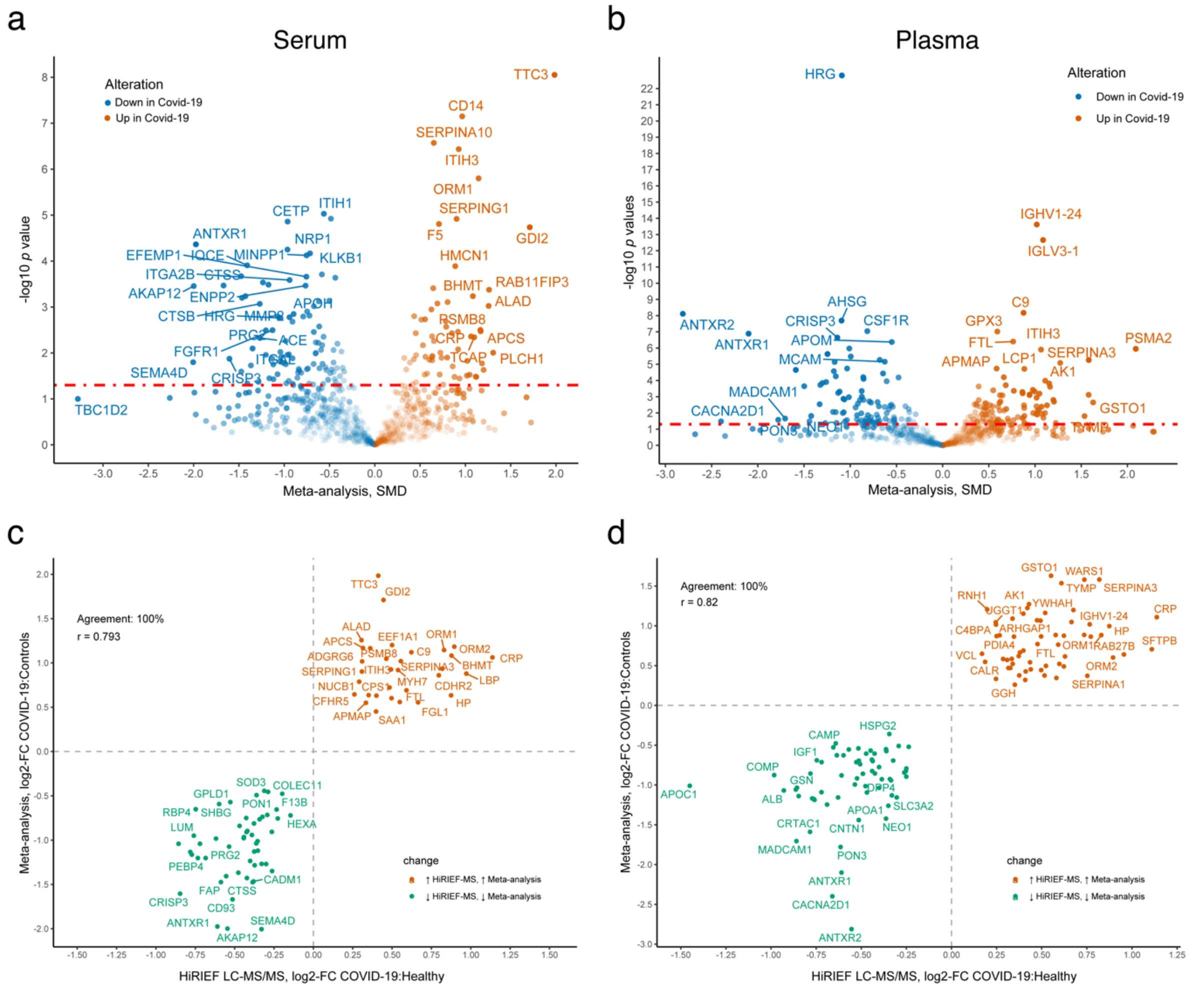

Figure S18. Meta-analysis stratified according to sample type. Volcano plots depicting the SMD as estimated with proteomics studies performed on serum (**a.**) and plasma samples (**b.**). The agreement with the estimates of HiRIEF LC-MS/MS for proteins that were statistically altered was high, regardless of whether the meta-analysis was based on proteomics studies profiling serum (**c.**) or plasma (**d.**) samples.

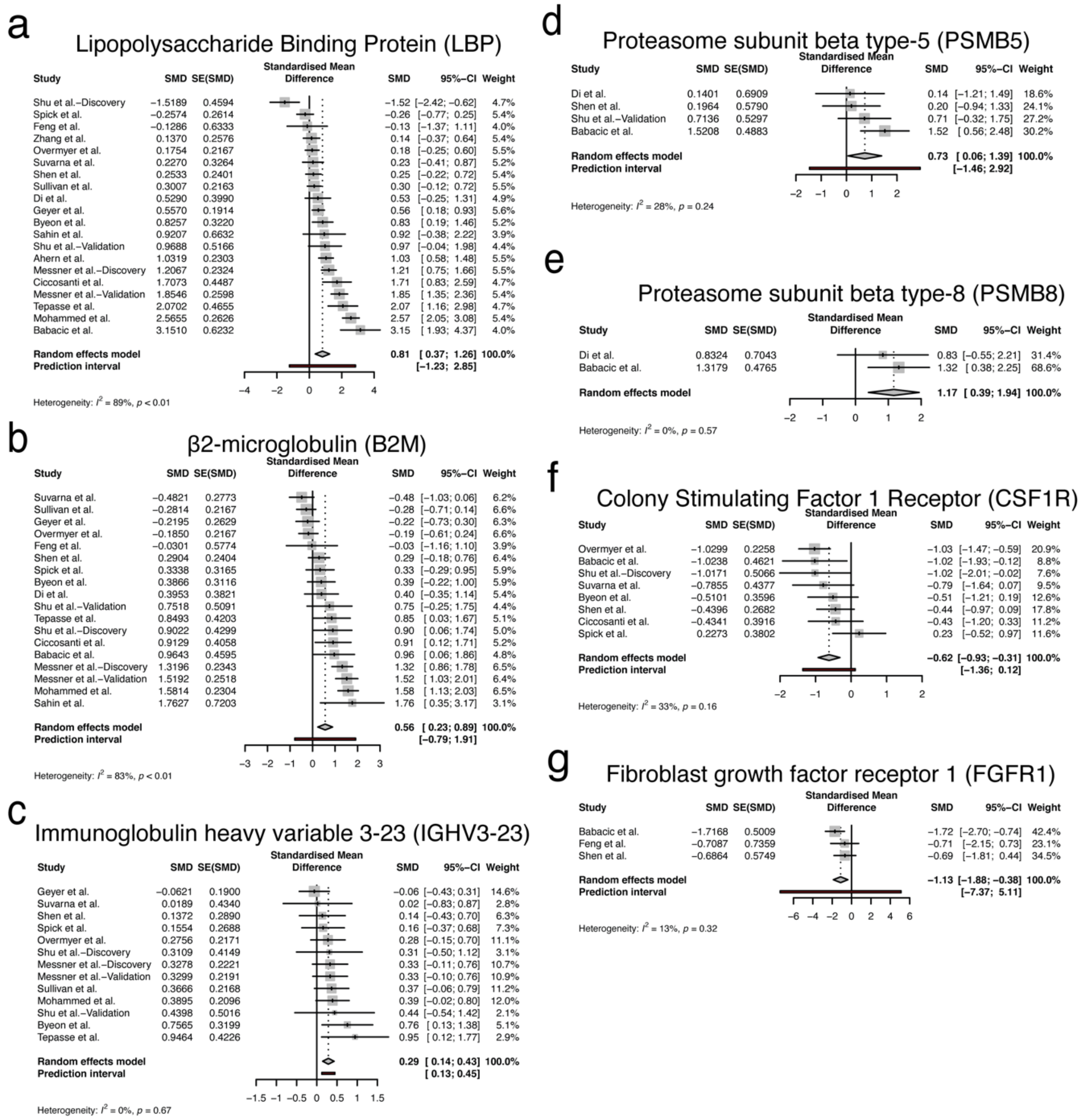

Figure S19. Forest plots of standardised mean difference (SMD) meta-analysis for selected proteins: The forest plots represent the SMD estimates (middle vertical line of the box), with 95% CI (horizontal line), for the selected protein in each study cohort in which it was quantified in at least three COVID-19 patients and at least three PCR-negative controls. SMD > 0 = elevated levels, SMD < 0 = decreased levels, in COVID-19 patients as compared to PCR-negative controls. The summary SMD is represented with a diamond shape, where the width corresponds to the 95% CI. The vertical dashed line represents the summary SMD; the estimates to the right and left of this line means that the studies have estimated a larger and smaller effect, respectively. Numeric values of the estimates, with 95% CI and weights, are given to the right of the plot. The prediction interval includes the interval in which estimates from future studies are expected. **a. Lipopolysaccharide Binding Protein (LBP)**, an acute

phase protein, generally considered as a marker of gut leakage, which is questionable; **b. β2-microglobulin (B2M)**, an MHCI class molecule, important for antigen presentation. Calnexin binds this protein; **c. Immunoglobulin heavy variable 3-23 (IGHV3-23)**, a clonotype which might be required for antibodies targeting SARS-CoV-2; **d-e. Proteasomal proteins PSMB5 and PSMB8** described only by us as upregulated in COVID-19, whereas the remaining studies showed no change. The summary SMD estimate in the meta-analysis confirms our finding; **f-g. Decrease in soluble blood levels of membrane receptors CSF1R and FGFR1** had varying statistical significance in the underlying studies, but the meta-analysis showed their decrease in the blood of COVID-19 patients.

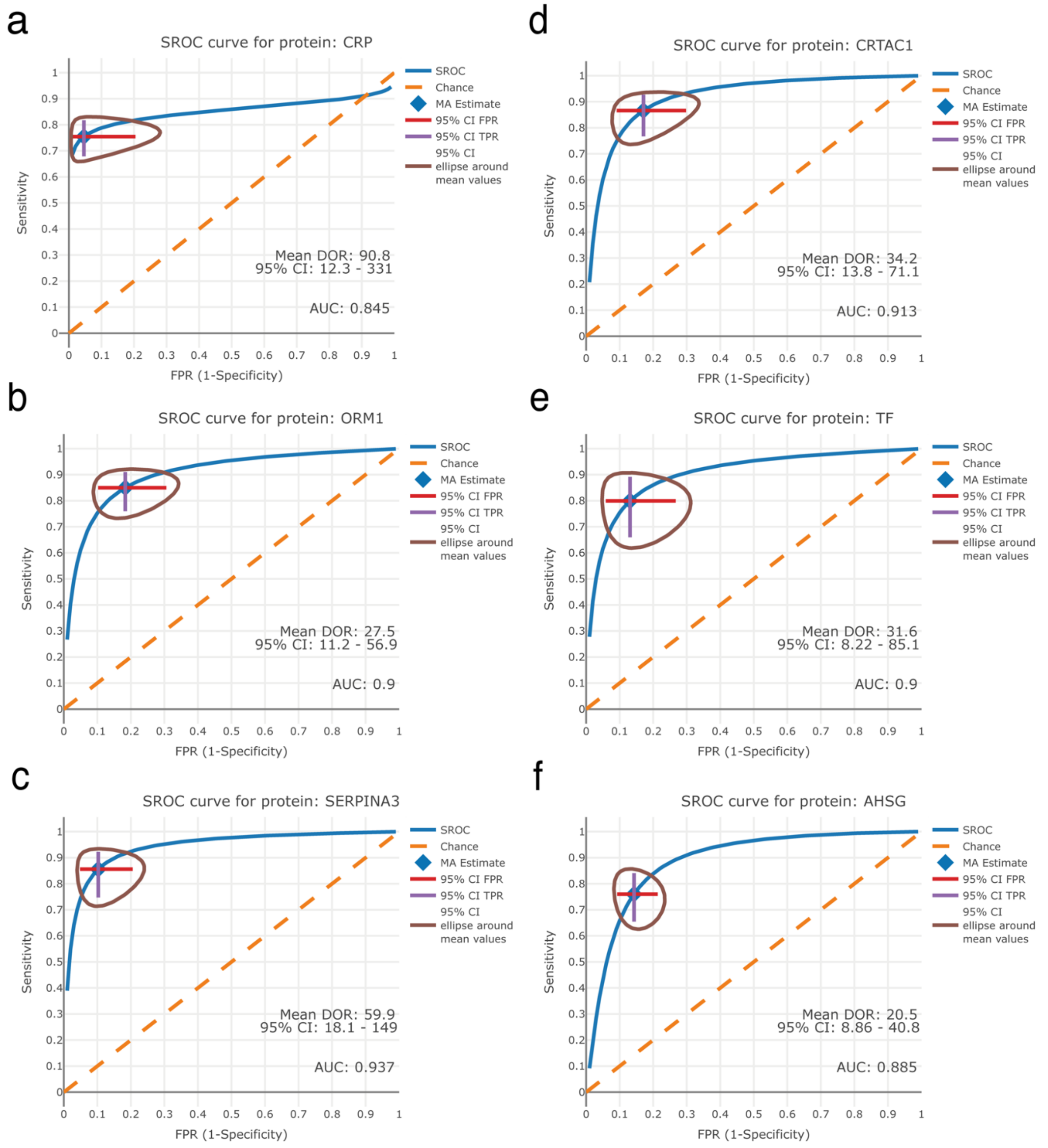

Figure S20. Plots of SROC meta-analysis for selected proteins with best AUC performance in comparison to CRP (a.): b. ORM1; c. SERPINA3; d. CRTAC1; e. TF; f. AHSG. The plots present the SROC curves, with false positive rate (1-specificity, x axis) and sensitivity (y axis). The diamond shape represents the mean sensitivity and 1-specificity estimates, with 95% confidence intervals (CI) for each as lines. The mean diagnostic odds ratios (DOR) with 95% CI and area under the curve (AUC) with 95% CI are also annotated on the plot.
