# Supplementary material for: Comprehensive proteomics and meta-analysis of COVID-19 host response
Source: Nat Commun. 2023 Sep 22;14:5921. doi: 10.1038/s41467-023-41159-z (PMC10516886; doi:10.1038/s41467-023-41159-z)
Supplement: Supplementary file 3 — Description of Additional Supplementary Files [file 41467_2023_41159_MOESM3_ESM.pdf]

# <u>Description of Additional Supplementary Files</u>

**Supplementary dataset 1.** Proteins identified by HiRIEF LC-MS/MS in comparison to PEA and SOMAscan analyses.

**Supplementary dataset 2.** Difference in protein serum levels between COVID-19 patients and healthy controls, two-sided t test.

**Supplementary dataset 3.** Comparison between alterations in soluble blood protein levels quantified by HiRIEF LC-MS/MS to estimates by PEA and SOMAscan. **a.** All differentially altered soluble blood proteins based on HiRIEF LC-MS/MS analyses annotated for tissue enrichment based on the Human Protein Atlas and whether they have been reported as altered by PEA and SOMAscan analyses; **b.** Soluble blood proteins identified as altered by HiRIEF LC-MS/MS in comparison to previous analyses with PEA; **c.** Soluble blood proteins identified as altered by HiRIEF LC-MS/MS in comparison to previous analyses with SOMAscan.

**Supplementary dataset 4.** Difference in protein serum levels between COVID-19 patients and healthy controls, estimated with a limma model, adjusted for age, sex, and comorbidities.

**Supplementary dataset 5.** Difference in protein levels in Calu-3 cell lines infected with SARS-CoV-2 in comparison to non-infected Calu-3 cell line, two-sided t test: **a.** 3 days after infection; **b.** 7 days after infection.

**Supplementary dataset 6.** Serum proteins' correlation matrix, containing Spearman's correlation coefficients between serum proteins.

**Supplementary dataset 7.** Matrix of proteins with altered levels in the serum as analysed by HiRIEF LC-MS/MS in comparison to the alterations observed in Calu-3 cell lines 3- and 7- days after SARS-CoV-2 infection and previous proteomic analyses of organs from patients deceased due to COVID-19.

**Supplementary dataset 8.** Matrix of proteins with altered levels in the serum as analysed by HiRIEF LC-MS/MS that had a consistent alteration in the same direction in Calu-3 cell lines 3- and/or 7- days after SARS-CoV-2 infection and/or at least one other organ in previous proteomic analyses of organs from patients deceased due to COVID-19.

**Supplementary dataset 9.** GSEA results, permutation test. **a.** COVID-19 organ-associated protein sets; **b.** Filtered COVID-19 organ-associated protein sets; **c.** MSiGDb hallmark gene sets; d. KEGG gene sets; e. MSiGDb REACTOME gene sets.

**Supplementary dataset 10.** Correlation between protein levels and clinical and anti-SARS-CoV-2 immune response markers.

**Supplementary dataset 11.** Phosphoproteomics results: **a.** Phosphopeptides altered in the serum of COVID-19 patients in comparison to healthy controls, two-sided t test; **b.** Phosphopeptides altered 3 days after SARS-CoV-2 infection in Calu-3 cell line, compared to non-infected controls; **c.** Phosphopeptides altered 7 days after SARS-CoV-2 infection in Calu-3 cell lines, compared to non-infected controls.

**Supplementary dataset 12.** Studies identified in the systematic review that were included in the meta-analysis.

**Supplementary dataset 13.** Results from the SMD meta-analysis: **a.** All proteins analysed in all studies; **b.** Stratified analyses of proteins identified in studies that used plasma as a sample; **c.** Stratified analyses of proteins identified in studies that used serum as a sample.

**Supplementary dataset 14.** Results from the SROC meta-analyses: **a.** All proteins analysed in all studies; **b.** Sensitivity analyses of the preference for sensitivity and specificity of the underlying studies.

**Supplementary dataset 15.** Gradient length per HiRIEF fraction.
