# Supplementary material for: Comprehensive proteomics and meta-analysis of COVID-19 host response
Source: Nat Commun. 2023 Sep 22;14:5921. doi: 10.1038/s41467-023-41159-z (PMC10516886; doi:10.1038/s41467-023-41159-z)
Supplement: Supplementary file 19 — Reporting Summary [file 41467_2023_41159_MOESM19_ESM.pdf]

nature portfolio

Corresponding author(s): Haris Babačić, Maria Pernemalm

Last updated by author(s): Aug 3, 2023

Reporting Summary

Nature Portfolio wishes to improve the reproducibility of the work that we publish. This form provides structure for consistency and transparency in reporting. For further information on Nature Portfolio policies, see our Editorial Policies and the Editorial Policy Checklist.

Statistics

For all statistical analyses, confirm that the following items are present in the figure legend, table legend, main text, or Methods section.

n/a	Confirmed	
☐	☒	The exact sample size (*n*) for each experimental group/condition, given as a discrete number and unit of measurement
☐	☒	A statement on whether measurements were taken from distinct samples or whether the same sample was measured repeatedly
☐	☒	The statistical test(s) used AND whether they are one- or two-sided<br>*Only common tests should be described solely by name; describe more complex techniques in the Methods section.*
☐	☒	A description of all covariates tested
☐	☒	A description of any assumptions or corrections, such as tests of normality and adjustment for multiple comparisons
☐	☒	A full description of the statistical parameters including central tendency (e.g. means) or other basic estimates (e.g. regression coefficient) AND variation (e.g. standard deviation) or associated estimates of uncertainty (e.g. confidence intervals)
☐	☒	For null hypothesis testing, the test statistic (e.g. *F*, *t*, *r*) with confidence intervals, effect sizes, degrees of freedom and *P* value noted<br>*Give P values as exact values whenever suitable.*
☒	☐	For Bayesian analysis, information on the choice of priors and Markov chain Monte Carlo settings
☒	☐	For hierarchical and complex designs, identification of the appropriate level for tests and full reporting of outcomes
☐	☒	Estimates of effect sizes (e.g. Cohen's *d*, Pearson's *r*), indicating how they were calculated

Our web collection on statistics for biologists contains articles on many of the points above.

Software and code

Policy information about availability of computer code

Data collection	The proteomics data were obtained by using our proteomics workflow (https://github.com/lehtiolab/ddamsproteomics, v.2.7), which was run with Nextflow (v.20.01.0). Orbitrap raw MS/MS files were converted to mzML format using msConvert from the ProteoWizard tool suite (v.3.0.21229). Spectra were searched using MSGF+ search engine (v2020.03.14) and Percolator (v3.04.0) for Percolator target-decoy scoring.
Data analysis	All analyses were performed in R v.4.2.2, and the figures assembled in Adobe Illustrator 2023 (v.24.0.1). The graphical abstract was created with Biorender (cloud-based software, last version accessed 15. July 2023). The following R packages were used in data processing, analysis, and visualisation: tidyverse (v.2.0.0), ggplot2 (v.3.4.1), matrixStats (v.0.63.0), VennDiagram (v.1.7.3), ggrepel (v.0.9.3), dplyr (v.1.1.0), factoextra (v.1.0.7), FactoMineR (v.2.7), ggfortify (v.0.4.15), cluster (v.2.1.4), gplots (v.3.1.3), RColorBrewer (v.1.1-3), ComplexHeatmap (v.2.14.0), readxl (v.1.4.2), ggpubr (v.0.6.0), msigdbr (v.7.5.1), grid (v.4.2.2), limma (v.3.54.2), ggven (v.0.1.9), circlize (v.0.4.15), org.Hs.eg.db (v.3.16.0), clusterProfiler (v.4.6.2), enrichplot (v.1.18.3), WGCNA (v.1.72-1), stringr (v.1.7.12), seqinr (v.4.2-30), DEqMS (v.1.16.0), cowplot (v.1.1.1), biomaRt (v.2.54.0), bioseq (v.0.1.4), cutpointr (v.1.1.2), pROC (v.1.18.0), mada (v.0.5.11), reshape2 (v.1.4.4), esc (v.0.5.1), dmetar (v.0.0.9000), meta (v.6.2-1), ggforce (v.0.4.1), plotly (v.4.10.1), ggpmisc (v.0.5.2), scales (v.1.2.1), ggsci (3.0.0), lme4 (v.1.1-32), kableExtra (v.1.3.4), pacman (v.0.5.1), and car (v.3.1-1).

For manuscripts utilizing custom algorithms or software that are central to the research but not yet described in published literature, software must be made available to editors and reviewers. We strongly encourage code deposition in a community repository (e.g. GitHub). See the Nature Portfolio guidelines for submitting code & software for further information.

Data

Policy information about availability of data

All manuscripts must include a data availability statement. This statement should provide the following information, where applicable:

- Accession codes, unique identifiers, or web links for publicly available datasets
- A description of any restrictions on data availability
- For clinical datasets or third party data, please ensure that the statement adheres to our policy

The personal data are not publicly available due to them containing information that could compromise research participant privacy. All other data are provided in the article and its Supplementary files or from the corresponding authors upon request. Source data are provided with this paper.
All results described in the manuscript are presented in main or supplementary figures or tables. The gene sets from the MSigDB are available for download in R with the msigdbr package (v.7.5.1). The Ensembl105 human protein database is available for download from https://www.ensembl.org/. The phosphosites are curated in the PhosphoSitePlus database, available at https://www.phosphosite.org/. All results from the meta-analysis are available as a shiny app resource at Https://doi.org/10.17044/scilifelab.22293148. Raw and processed MS data are deposited via ProteomeXchange to the PRIDE database, with accession codes PXD037486, PXD037451, and PXD040982.

Research involving human participants, their data, or biological material

Policy information about studies with human participants or human data. See also policy information about sex, gender (identity/presentation), and sexual orientation and race, ethnicity and racism.

Reporting on sex and gender	Sex was included as a covariate in multivariate differential analyses. We did not collect information on gender.
Reporting on race, ethnicity, or other socially relevant groupings	We did not analyse race and ethnicity due to lack of information. However, in the meta-analysis data collected from different countries and different populations have been analysed.
Population characteristics	Age and corticosteroid treatment were used in multivariate differential analyses.
Recruitment	Twenty hospitalised SARS-CoV-2 PCR-confirmed patients from Karolinska University Hospital in Stockholm, Sweden, were sequentially recruited in the study in April 2020. Inclusion criteria were ongoing acute COVID-19 infection and self-declared healthy individuals, for cases and controls, respectively; exclusion criteria for both groups were known immunosuppression or immunosuppressive disease. Immunosupressed individuals were excluded because this could have confounded the analyses - such individuals might not be representative of a host response to SARS-CoV-2. In the HiRIEF LC-MS/MS analyses the small sample size and the exclusion of immunosupressed individuals have likely limited the analyses in generalising the host response to SARS-CoV-2 infection. Furthermore, the analyses were performed on hospitalised patients, thus limiting the analysis of host response to patients with moderate to severe COVID-19.<br><br>In the meta analysis, we included all the data from global mass-spectrometry proteomics studies performed on adult humans, not restricting for age, sex, COVID-19 severity, or population origin. We excluded proteomic analyses of children and pregnant women due to the large physiological differences in these populations compared to adult humans. This has limited the generalisability of the identified alterations to adult humans, which cannot be extended to children and pregnant women.
Ethics oversight	The study was approved by the Regional Ethical Review Board in Stockholm, Sweden and by the Swedish Ethical Review Authority, and is in accordance with the Declaration of Helsinki.

Note that full information on the approval of the study protocol must also be provided in the manuscript.

Field-specific reporting

Please select the one below that is the best fit for your research. If you are not sure, read the appropriate sections before making your selection.

☒ Life sciences ☐ Behavioural & social sciences ☐ Ecological, evolutionary & environmental sciences

For a reference copy of the document with all sections, see nature.com/documents/nr-reporting-summary-flat.pdf

Life sciences study design

All studies must disclose on these points even when the disclosure is negative.

Sample size	No a priori sample size calculation was performed. Considering the median value of the per-protein variance distribution, assuming a median pooled standard deviation of 0.256 in relative protein abundance, the study had a power of 80% to detect an effect size of 0.328 with a two-sided unpaired t test, at a significance level of alpha = 0.05.
Data exclusions	No samples were excluded. Proteins with >50% missing values were excluded from the analyses.
Replication	No replication was performed by us to reproduce the discovery of the alterations in the serum proteome. Instead, we have used public

Replication	datasets of affinity- and mass-spectrometry-based proteomics to validate and reproduce our findings.<br><br>The infection experiment was performed in biological replicates.
Randomization	The SARS-CoV-2 virus and mock treatment with UV-inactivated virus were randomly assigned to Calu-3 cell cultures.
Blinding	Blinding was not possible because the investigators had to know the nature of the samples to perform the experiments, design the TMT sets, and analyse the data. It is unlikely that the knowledge of the sample type during experiments and sample preparation has affected the identified proteome alterations because the samples coming from different conditions were processed in parallel.

Reporting for specific materials, systems and methods

We require information from authors about some types of materials, experimental systems and methods used in many studies. Here, indicate whether each material, system or method listed is relevant to your study. If you are not sure if a list item applies to your research, read the appropriate section before selecting a response.

Materials & experimental systems

n/a	Involved in the study
☒	☐ Antibodies
☐	☒ Eukaryotic cell lines
☒	☐ Palaeontology and archaeology
☒	☐ Animals and other organisms
☒	☐ Clinical data
☒	☐ Dual use research of concern
☒	☐ Plants

Methods

n/a	Involved in the study
☒	☐ ChIP-seq
☒	☐ Flow cytometry
☒	☐ MRI-based neuroimaging

Eukaryotic cell lines

Policy information about cell lines and Sex and Gender in Research

Cell line source(s)	Human lung adenocarcinoma Calu-3 cells (ATCC, HTB-55)
Authentication	Not authenticated
Mycoplasma contamination	Not tested for Mycoplasma
Commonly misidentified lines (See ICLAC register)	The cell line does not belong to commonly misidentified lines.
